# Supplementary material for: Comparative Clinical Characteristics, Laboratory Findings, and Outcomes of Hypoxemic and Non-Hypoxemic Patients Treated at a Makeshift COVID-19 Unit in Bangladesh: A Retrospective Chart Analysis
Source: J Clin Med. 2022 May 24;11(11):2968. doi: 10.3390/jcm11112968 (PMC9181205; doi:10.3390/jcm11112968)
Supplement: Supplementary file 1 [file jcm-11-02968-s001.zip › jcm-1666780-supplementary.pdf]

**Table S1**. Treatment and outcome profiles of COVID-19 affected in-patients.s

| Characteristics | Oxygen required (n=88) | Oxygen not required (n=119) |
|---|---|---|
| **Antibiotics** | | |
| Ceftriaxone | 80 (90.9) | 43 (36.1) |
| Ciprofloxacin | 74 (84.1) | 39 (32.8) |
| Meropenem | 35 (39.8) | 4 (3.4) |
| Tigecycline | 34 (38.6) | 4 (3.4) |
| **Antiviral** | | |
| Favipiravir | 6 (6.8) | 7 (5.9) |
| Remdesivir | 14 (15.9) | 01 (0.8) |
| **Antihistamine** | 13 (14.8) | 25 (21) |
| **Sedatives** | 33 (39.8) | 31 (29) |
| **Salbutamol inhaler** | 34 (39.5) | 26 (24.5) |
| **PPI** | 41 (47.1) | 30 (27.3) |
| **Long acting insulin** | 26 (30.6) | 6 (5.6) |
| **Short acting insulin** | 34 (40) | 10 (9.3) |
| **Complications** | 63 (71.6) | 0 |
| Heart failure | 20 (22.7) | 0 |
| Septic shock | 15 (17) | 0 |
| ARDS | 43 (48.9) | 0 |
| **Prognosis** | | |
| Discharge | 62 (70.5) | 116 (97.5) |
| Referral | 7 (8) | 3 (2.5) |
| Death | 19 (21.6) | 0 |
| **Hospital stay (days) (median, IQR)** | 9 (6, 14) | 8 (4, 14) |
